# The prevalence of depression and anxiety symptoms and their associated factors among patients with cancer in Qatar: A cross-sectional study

**DOI:** 10.5339/qmj.2025.4

**Published:** 2025-02-07

**Authors:** Majid Alabdulla, Shuja Reagu, Moza Alishaq, Noora Al Hammadi, Mohammed Hassan Elkordy, Hafedh Ghazouani, Ahmed H. Assar

**Affiliations:** ^1^Department of Psychiatry, Hamad Medical Corporation, Doha, Qatar; ^2^College of Medicine, Qatar University, Doha, Qatar; ^3^Corporate Quality Improvement and Patient Safety Department, Hamad Medical Corporation, Doha, Qatar; ^4^National Center for Cancer Care and Research, Hamad Medical Corporation, Doha, Qatar *Correspondence: Mohammed Hassan Elkordy. Email: mali51@hamad.qa

**Keywords:** Anxiety, cancer, depression, patients, Qatar, sociodemographic factors

## Abstract

**Background:**

Cancer is a significant global health challenge. One of the biggest health issues that cancer patients face is depression and anxiety. This has a significant impact on their quality of life and treatment outcomes.

**Aim:**

The aim of this study was to investigate the frequency of depression and anxiety among cancer patients in Qatar.

**Materials and methods:**

This study was a cross-sectional design using the Patient Health Questionnaire-9 (PHQ-9) and Generalized Anxiety Disorder-7 (GAD-7) scales. A total of 500 cancer patients were surveyed from the National Center for Cancer Care and Research in Doha.

**Results:**

The study found that a significant proportion of cancer patients suffered from depression, with an average PHQ-9 score indicating mild levels of severity. Depression was commonly categorized as mild, with a smaller percentage experiencing moderate, moderate-to-severe, or severe depression. Additionally, patients were predominantly anxious, as reflected by an average GAD-7 score, with most patients experiencing mild to moderate symptoms, while a few experienced moderate or severe anxiety. These findings highlight the significant prevalence of both depression and anxiety among cancer patients, pointing to the importance of comprehensive mental health support. Moreover, patients with advanced-stage cancer, those in their 40s and 60s, those undergoing radiotherapy or hormone therapy, and female patients were found to be more susceptible to depression and anxiety.

**Conclusion:**

Treatment of mental health issues is essential to enhancing the effectiveness of cancer treatment. Cancer patients can have a higher quality of life and better adherence to cancer treatments when mental illnesses such as depression and anxiety are identified and treated early. Furthermore, most patients reported having depression and anxiety, according to the study, which showed that these conditions were more common in Qatar than in other countries. Several demographic groups have been linked to higher rates of depression and anxiety, including women, middle-aged adults, people with stage IV cancer, and patients receiving therapies such as radiotherapy and chemotherapy.

## Introduction

Cancer is undoubtedly one of the greatest health challenges today and the second leading cause of death worldwide. Recent estimates suggest that a significant number of deaths have been reported. In 2018, this devastating disease tragically killed approximately 9.6 million people. The fact that it accounts for one in three deaths in developed countries is shocking.^1,2^

Qatar National Cancer Registry data shows that Qatar is not immune to this trend. This data analysis provides a clear picture of cancer prevalence in Qatar, with an increase of approximately 1,500 new cancer diagnoses per year. This number is likely to increase due to various lifestyle factors, such as increased life expectancy and an aging population. In 2019, the rate of this disease had increased to 17.97 cases per 100,000. This worrying statistic also implies a mortality rate of approximately 7%.^3^

If a person is diagnosed with cancer, they may be more vulnerable to mental disorders than healthy people due to the significant psychological impact. The disease can have a significant impact on mental health and manifest itself in a variety of ways. This phenomenon could be caused by the many difficulties that cancer brings. The sudden onset of cancer can cause patients to experience a range of intense emotions, including shock, disbelief, anger, depression, anxiety, and deep sadness.^4,5^

Approximately one-third of cancer patients suffer from mental disorders, the most common being depression. Failure to address these mental health issues can result in serious consequences such as lower treatment adherence, lower chances of survival, higher healthcare costs, and a worsening of the patient's overall quality of life.^6^ Literature from developed countries suggests that cancer patients have higher rates of depression and anxiety compared to the general population and that comorbidity with depression may lead to increased morbidity and worse cancer outcomes.^7,8^

Although depression and anxiety are the most common complications in cancer patients, they are often overlooked. Furthermore, the psychosocial needs of cancer patients, regardless of whether they have a mental illness history or not, are often ignored in cancer therapy, which mainly focuses on the treatment of somatic symptoms and side effects. Earlier detection and improved treatment of cancer make people with cancer live longer.^9,10^

Internationally, depression is prevalent among cancer patients. For example, it is estimated that 23.4% of cancer patients in China suffered from depression,^11^ as well as 48.7% in Pakistan,^12^ 17% in Australia,^13^ 38.2% in Greece,^14^ 46.5% in Saudi Arabia,^15^ 67.7% in Rwanda,^16^ 25% in Addis Ababa and Ethiopia,^17^ and 58.4% in Gondar had an epidemiology of depression.^18^ Numerous factors have been associated with depression in various studies, including type of cancer, female gender, old age, duration of cancer, and type of therapy. Additionally, hospitalized patients were more likely to experience depressive symptoms compared to outpatients.^19,20^ The majority of research on depression in cancer patients comes from the developed world, while only a few studies focused on patients in Qatar.

## Study Objective

The aim of this study was to investigate the frequency of depression and anxiety among cancer patients in Qatar. In addition, we analyze associated sociodemographic and clinical factors and identify variables that predict the severity of these diseases.

### Study hypotheses

**H1:** Depression and anxiety are prevalent among cancer patients at the National Center for Cancer Care and Research (NCCCR) in Doha.

**H2:** There is a significant difference between sociodemographic factors and the prevalence of anxiety and depression among cancer patients at the NCCCR in Doha.

**H3:** Severe depression and anxiety are more common in patients with advanced-stage cancer (stage IV) than in patients with early-stage cancer.

**H4:** Cancer patients are more likely to experience depression and anxiety after receiving chemotherapy or radiotherapy.

**H5:** Anxiety and depression symptoms are more common in cancer patients with a family history of the disease or with a past psychiatric illness**.**

**H6:** Anxiety and depression symptoms are more common in older patients than in younger patients.

**H7:** Anxiety and depression symptoms are more common in female patients than in male patients.

## Materials And Methods

### Study design

A comprehensive cross-sectional observational study was conducted in Doha, Qatar.

### Study period and area

The study was conducted at NCCCR outpatient clinic from February 11, 2023 to March 30, 2023.

### Description of study participants and samples

In the prevalence study, sample size guidelines from the World Health Organization were followed (Lwanga and Lemeshow, 1991).^21^ The random sampling method resulted in a 95% confidence level with a standard deviation of 0.5 and a margin of error of 5%. Therefore, based on these parameters, the minimum sample size of 347 participants per group was required. The target individuals of the survey were 18 years old. A total of 600 responses were collected.

Consequently, 67 were rejected, 33 were incomplete, and 26 required more information. The study involved 541 individuals, some of whom had non-cancerous hematological conditions. A total of 501 eligible individuals were analyzed and evaluated. The data collection process was optimized by enlisting the expertise of five skilled and experienced nursing researchers. They conducted survey interviews using cutting-edge state-of-the-art notebook computers and electronic pens, all seamlessly integrated with computer touch-screen technology. Completing the questionnaire only took 10 minutes. Depending on the participant's preferred language, interviews were conducted in both Arabic and English. A translated version of the questionnaire was prepared by experts from mental health services and corporate quality improvement services, and administered to participants who indicated that they preferred Arabic. The translation process used a forward–backward approach to ensure accuracy. This initially required a bilingual specialist to translate everything from English to Arabic, and then another translator to translate everything back into English. A team of language specialists and medical professionals examined the discrepancies to ensure conceptual consistency. Furthermore, a qualified nurse conducted the interview with the illiterate participants verbally. The team reviewed and cross-referenced all data provided to ensure accuracy. All records with incomplete data and missing information were removed. Data were meticulously documented using an Excel spreadsheet and coding techniques to ensure accuracy. An analysis of the integrity of the data was conducted to determine whether it was suitable for further research.

### Data collection tool

The study was based on a relational screening model using the Patient Health Questionnaire-9 (PHQ-9) and the Generalized Anxiety Disorder-7 (GAD-7).

#### PHQ-9

The PHQ-9 is a self-report tool that can be used to identify depression and the severity of its symptoms.^21^ The range of possible responses was from “not at all” to “nearly every day”. The total questionnaire scores ranged from zero (0) to 27, with higher scores indicating more severe depressive symptoms. The PHQ-9 consisted of nine main questions and one supplementary question. The answer to each question was scored from 0 to 3 depending on the frequency of a given symptom in the past two weeks (three most common symptoms). The maximum score was 27, indicating the highest severity of depressive symptoms. The norm is a score of < 5. The range of 5–9 indicates mild depression, 10–14 indicates moderate depression, 15–19 indicates moderately severe depression, and 20–27 indicates severe depression.^22^

#### GAD-7

The GAD-7 is a seven-item self-report instrument used to assess the severity of GAD and general anxiety symptoms.^23^ Each item asks the individual to rate the severity of their symptoms over the past two weeks using a four-point Likert scale, with possible responses ranging from “Not at all” to “Nearly every day”. The total score ranges from zero (0) to 21, with higher scores indicating more severe anxiety symptoms. In the present study, cut-off points of 5, 10, and 15 were used as indicators of mild, moderate, and severe anxiety, as proposed by the scale authors.

### Variables

#### Dependent variables

The severity of depression (dependent variable) is defined by a score of 5. The range of 5–9 indicates mild depression, 10–14 indicates moderate depression, 15–19 indicates moderately severe depression, and 20–27 indicates severe depression. On the contrary, anxiety levels were measured using cut-off points of 5, 10, and 15 as indicators of mild anxiety, moderate anxiety, and severe anxiety, respectively.

#### Independent variables

The study analyzed demographic data by categorizing age into 20-year intervals. Gender was distinguished between males and females, while ethnicity was classified as Arab or non-Arab. Based on the clinical findings, cancer types were divided into hematologic malignancies and solid tumors. The study also investigated cancer stages I, II, III, and IV. Additionally, the marital status of the participants was analyzed and categorized them as single, married, or divorced.

Furthermore, the job function of employers was divided into 12 different categories, ranging from housewives and healthcare professionals to engineers and computer workers, drivers, business professionals, educators, office staff, clerical support workers, unskilled laborers, and retired personnel. Furthermore, individuals were divided into four groups based on their education level: illiterate, primary, secondary, and tertiary. Moreover, the duration since cancer diagnosis was classified into three groups: less than 12 months, between 12 and 60 months, and above 60 months. Additionally, “Yes” was recorded for specific variables when respondents confirmed a history of psychiatric illness, a family history of psychiatric illness or cancer, comorbidities, a COVID-19 infection, or a previous hospitalization due to COVID-19. Finally, three distinct stages were identified for the treatment phase: maintenance of a disease-free state, relapse or progression, and newly diagnosed cases.

### Statistical analysis

Descriptive statistics were used to evaluate the demographic characteristics, medical history, and levels of depression and anxiety among the participants. Frequencies and percentages were reported for categorical variables and means and standard deviations for continuous variables. Additionally, an inferential analysis was performed to evaluate symptom severity in different groups using various statistical tests, including parametric (t-tests and ANOVA) and non-parametric (Kruskal–Wallis and Mann–Whitney U) tests. The second step involved examining the relationship between depression levels, anxiety levels, and other variables using the chi-square test and Fisher's exact test. In addition, the biserial correlation coefficient was used to investigate the association between binary and ordinal variables related to the frequency of depression and anxiety disorders.

The prevalence of depression and anxiety symptoms in patients was measured by following the authors’ recommendations for the PHQ-9 and GAD-7 cut-off points, and then dividing the total number of patients by those who met the criteria. Upon further review, only 3% of the data for these crucial outcomes was found to be missing. To ensure accuracy, various imputations were used to estimate and replace the missing values. Additionally, the analysis involved the use of logistic regression for bidirectional analysis. The first attempt was to model an ordinal variable using quantitative and qualitative explanatory variables to determine whether demographic, social, and clinical factors influence anxiety and depression. A score of 0 means that there is neither anxiety nor depression, while a value of 1 means that both are present. To create accurate reference groups, patients with depression and anxiety were examined.

Additionally, potential risk factors and predictors of anxiety and depression symptoms in cancer patients were examined using ordinal regression to include odds ratios (OR) with 95% confidence intervals (CIs). The results were statistically significant, with a *p* value of less than 0.05. Statistical analysis was performed using Stata software version 15.1.

### Ethical considerations

The study was approved by the Research Ethics Committee of Hamad Medical Corporation under protocol number MRC-01-21-267 on February 21, 2022. Participants were informed about the purpose of the study, confidentiality was assured, and participation was voluntary. All responses were anonymous and used for research purposes only. The study followed the STROBE (Strengthening the Reporting of Observational Studies in Epidemiology) guidelines for comprehensive and transparent reporting.

## Results

Table 1 provides a detailed summary of the demographic, social, and clinical characteristics of the study participants. A total of 501 participants took part in the study, 50.6% of whom were male. The participants were on average 47.9 years old (SD = 11.4 years), and 74% were of Arab descent. According to the study, 19.76% of the participants were diagnosed with hematological malignancies. The prevalence of other types of cancer was reported as follows: colorectal cancer (9.38%), breast cancer (8.98%), and pancreatic cancer (12.18%). Moreover, 43 cancer cases were not specified, of which 12 had no further information. In addition, a significant proportion of participants (41.12%) had coexisting medical conditions. Furthermore, 83% of the survey respondents were single, 14% were married, 1% was divorced, and 13% were widowed. In addition, 41% of the respondents were housewives, 39% worked in the service sector, 15% were unemployed, 14% worked in the healthcare sector, and 12% worked in the technology sector. Regarding cancer stages, 25% of the participants were at stage I, 33% at stage II, 55% at stage III, 24% at stage IV, and 4% had an unknown stage. The treatments used were as follows: 5.0% radiotherapy, 21.4% chemotherapy, 9.3% surgery, 12.7% radiotherapy and chemotherapy, 11.3% chemotherapy and surgery, 29% radiotherapy and surgery, and 11.3% comprehensive treatment with chemotherapy, radiotherapy, and surgery. Remarkably, a fraction of approximately 10% of the participants received alternative treatment approaches. These included innovative treatments such as immunotherapy, hormone therapy, and targeted therapy. Nearly half of the participants had completed tertiary education (48.5%), whereas illiteracy was observed in only 5.1% of the study participants, followed by secondary education in 38.32% and primary education in 7.98%. On average, cancer was diagnosed 18 months after the first self-reported symptoms (SD = 17.8 months) and treated for 12.5 months (SD = 20.5 months). In addition, 33.13% of the participants had a family history of cancer, 3.19% had a family history of mental illness, and 2.40% had a mental illness themselves. Of the participants, 21% were infected with COVID-19 and 30% were hospitalized due to COVID-19 symptoms. Most study participants (52.31%) were in remission or disease-free. Only 9.18% were recently diagnosed with cancer, but a significant percentage (38.52%) experienced disease relapse or progression.

Table 2 shows that of the 500 cancer patients, 378 suffered from depression. The overall prevalence rate for depression was 75.05% (95% CI: 62.00, 84.37), with an average PHQ-9 score of 3.28 ± 3.90 points. Of these, 20% suffered from minimal depression, 40% from mild depression, 8.18% from moderate depression, 1.97% from moderately severe depression, and 7.98% from severe depression. Additionally, of the 500 participants, 352 reported anxiety symptoms. Overall, 70.25% of the population was anxious (95% CI: 59.12, 74.85), and their average GAD-7 score was 3.17 ± 3.41 points. Furthermore, 15.56%, 48.30%, 4.19%, and 2.19% of the patients were categorized as mild, mild-to-moderate, moderate, and severe anxiety, respectively.

As shown in Table 3, a point-biserial correlation coefficient (*r*_pbi_) was calculated to examine the relationship between sociodemographic variables and symptoms of depression and anxiety. Table 3 shows that the study found a weak but significant positive correlation between depression symptoms and cancer stage (*r*_pbi_ = 0.05, *p* < 0.05), followed by a family history of cancer (*r*_pbi_ = 0.04, *p* < 0.04) and having undergone hormone therapy (*r*_pbi_ = 0.08, *p* < 0.05). The study also found a weak positive correlation between anxiety severity and psychiatric history (*r*_pbi_ = 0.08, *p* < 0.05), radiotherapy (*r*_pbi_ = 0.10, *p* < 0.05), and gender (*r*_pbi_ = 0.12, *p* < 0.05).

Table 4 compares the participants’ depression and anxiety scores using ANOVA and Student's t-test. It was found that certain groups of participants had higher scores on the PHQ-9 depression scale than others. These groups include individuals employed in the driving profession (with a mean score of 8.0 [SD: 4.0]), those with a primary school education (with a mean score of 8.1 [SD: 6.0]), participants with psychiatric illnesses (with a mean score of 7.8 [SD: 5.8]), individuals who underwent chemotherapy (with a mean score of 5.3 [SD: 4.4]), participants with stage I cancer (with a mean score of 5.6 [SD: 4.3]), and those who received radiotherapy (with a mean score of 6.9 [SD: 4.4]). During examination, we found that anxiety levels varied significantly between different groups. According to the GAD-7 score, some groups showed higher levels of anxiety than others. Among all participants, those in the driving profession had the highest mean GAD-7 score of 6.5 (SD: 5.5). Additionally, individuals who had completed only primary school had a mean GAD-7 score of 6.8 (SD: 5.2), while those with mental illness had a mean GAD-7 score of 6.2 (SD: 4.7). Participants with a family history of mental illness had a mean GAD-7 score of 6.5 (SD: 3.9). Those who received radiotherapy or hormone therapy achieved a mean score of 5.6 (SD: 4.6) and 5.6 (SD: 4.2), respectively.

Table 5 shows the risk factors for depression and anxiety in cancer patients. The analysis showed that women were 59% more likely to develop depression than men. Individuals between the ages of 40 and 60 years had a 143% higher risk of developing depression. Retirees had a 24% lower risk of depression than people in other professions, while office workers had a 34% lower risk. Completing secondary school reduced the risk of depression by 0.22 times. People with comorbidities were 63% more likely to develop depression, while those diagnosed with stage 4 cancer were 2.10 times more likely to develop depression. The analysis also found that women were 66% more likely to develop anxiety than men. Married people were 84% less likely to suffer from anxiety disorders. Service workers were 72% more likely to suffer from anxiety disorders, while education workers were 1.39 times more likely to suffer from anxiety disorders. Participants with a college degree were 1.47 times more likely to suffer from anxiety disorders. These determinants had a significant impact on the likelihood of developing anxiety. Additionally, participants with comorbid medical conditions had a 63% increased risk of developing anxiety disorders. People diagnosed with stage IV cancer were 0.96 times more likely to develop anxiety. However, people in the relapse phase were 0.50 times less likely to experience anxiety. However, those who underwent radiological treatment were 0.51 times more likely to suffer from anxiety.

## Discussion

Mental disorders such as depression and anxiety can have a significant impact on the health of cancer patients. Oncology settings mainly deal with this problem. However, there is a lack of information about these disorders and their causes in Qatar. A total of 371 cancer patients who were treated as outpatients took part in the study. The study found that 75% suffered from anxiety, while 70% experienced depression. These findings were consistent across all cancer types. Mental health conditions can have a significant impact on the well-being of cancer patients. This study investigates how demographics, clinical conditions, and behavior influence anxiety and depression levels.

This study shows that depression is significantly more common in Qatar than in other countries. Analysis of depression revealed that most participants (75.5%) reported being clinically depressed. Most cases were associated with milder symptoms, accounting for 40.1% or more of the total number of depression cases. The prevalence rate of depression in this study was significantly higher than the rates found in two previous comprehensive studies examining depression prevalence among cancer patients. The first study by Krebber et al. reviewed 211 articles, which found that depression rates varied from 8% to 24% depending on the type of cancer, the stage of the disease, and the diagnostic method used.^24^ The second study, which examined 66 studies, estimated the prevalence of depression among cancer patients at 16.3%.^25^

On the contrary, studies have shown that depression is more common among cancer patients in Qatar compared to other countries. This research found a prevalence rate higher than that of most studies, with rates ranging from 43% in Pakistan (95% CI: 26%–64%)^11^ to 39% in Italy (95% CI: 25%–57%),^26^ 13% in Taiwan (95% CI: 4%–36%),^27^ and 14% in Germany (95% CI: 10%–19%).^28^ However, this study confirms that cancer patients often experience psychological or psychiatric complications, which may be influenced by cultural differences, tools used to measure depression, and the stressful outpatient interview environment.

In this study, which was conducted in an outpatient setting, found notable differences from the findings of Walker et al. Their research reported varying rates of depression among different patient groups, ranging from 5% to 16% in outpatients, 4% to 14% in inpatients, 4% to 11% in mixed-outpatient /inpatient, and 7% to 49% in patients receiving palliative care.^29^

This research revealed that various factors have a large influence on higher depression scores. Among these factors, the type of breast cancer stands out. Previous studies have clearly shown that the occurrence of depression poses a significant risk for individuals diagnosed with certain types of breast cancer.^30^ Furthermore, it has been found that being a driver can have a profound effect on depression, as demonstrated by a recent study.^31^ This study found a significant and alarming 84% increase in reported cases of depression among drivers since 2017. This research also revealed specific factors that increase the likelihood of developing depression. These include individuals who have only completed primary school education, those suffering from psychiatric disorders, and those who have undergone chemotherapy.^32^ The increased occurrence of chemotherapy as a primary form of treatment may be due to the fact that patients with terminal and unfavorable prognoses have chosen it.^33^

This research has provided important insights into the relationship between depression severity and various factors, including cancer stage, hormone therapy, and family history. The results suggest that specific groups of individuals, such as women, those with comorbidities, middle-aged adults (between 40 and 60 years old),^34^ and those with stage IV cancer,^35^ are particularly susceptible to developing depression. It is intriguing to note that the findings are consistent with previous studies conducted in Iran, particularly in patients with stage IV cancer and individuals who have undergone radiotherapy. Moreover, these factors can significantly contribute to the prevalence of depression, likely due to the physical changes resulting from the illness and its treatments. This has been supported by previous research highlighting the profound impact of images on self-image and sexual drive, both of which can have detrimental effects on mental health, particularly with regard to depression.^9^ Furthermore, there are several possible reasons for the occurrence of psychological disorders in such patients, including disease-related complications such as pain, disfigurement, family dependence or breakdown, social and financial setbacks, and even mortality.^36^

This study revealed a remarkable finding concerning the anxiety levels of the participants. Interestingly, the results showed that 70.25% of individuals suffered from severe anxiety, while 48.50% had mild anxiety. These findings highlight the alarming prevalence of anxiety disorders among cancer patients, surpassing global research in this area. Remarkably, observations indicate that anxiety rates are strikingly high compared to other countries. The report exhaustively examined anxiety rates in various countries, revealing that Germany reported a rate of 12.3%,^37^ Iran 46%,^38^ China 43.5%,^39^ India 46.91%,^40^ and Sudan 26.7%.^41^ In addition, this study revealed an intriguing finding that surpassed the insights of an enlightening meta-analysis conducted by Mitchell. In that study, anxiety disorders were found to affect approximately 10% of a large population of 10,071 cancer patients from 14 different countries, as well as 4,007 cancer patients from seven different countries.^42^ To explain this striking difference, several contributing factors were identified. These included variations in cancer types, screening methods, sociodemographic elements, and even depression severity. After a thorough analysis, several key factors contributing to anxiety among cancer patients were identified. These included the type of cancer, clinical characteristics, sociodemographic elements, and severity of depression. Extensive investigation and analysis are required to better understand these underlying factors. This research found an association between anxiety disorders and factors such as a history of mental health issues, radiotherapy, and gender disparities.^43^

Moreover, this research showed that specific variables were associated with elevated scores on factors that directly influenced anxiety. These variables included a personal history of mental illness, driving profession, primary school education, family history of mental illness, female gender, and previous radiotherapy and/or hormone therapy. At the same time, another research suggested that education level was not associated with anxiety levels.^44^

This research revealed a variety of risk factors that significantly increased the likelihood of anxiety in cancer patients. Advanced-stage cancer is associated with various risk factors, including a personal history of mental illness, previous exposure to radiotherapy or hormone therapy, and being female.^45^ Furthermore, a study by Amine, et al(2021). revealed a strong connection between anxiety and being female, levels of physical activity, and limitations in social roles. These findings are supported by a recent study by Huppert and his team, emphasizing the importance of considering these factors in treatment planning in order to provide the best possible care to patients with advanced stages of cancer.^46^ While it is true that chemotherapy or other cancer treatments can lead to a decrease in appetite and intense emotions such as sadness, anger, anorexia, and anxiety, it is crucial to acknowledge that chemotherapy remains a highly effective treatment that significantly improves survival rates.^25^ To further confirm this point, a study involving oncology patients conducted by Al Ishaq et al. (2023) demonstrated that anxiety was predicted by factors such as being female, impaired physical activity, and poor social role functioning, while fatigue predicted depression. These findings further strengthen the evidence for the effectiveness of chemotherapy.^47^

Oncology patients should be closely monitored for symptoms of depression and anxiety. There is evidence that providing psychosocial support reduces depression, anxiety, and pain in this population.^48^ Early detection of anxiety and depression in this population can help provide effective treatment interventions, including education, support, psychotherapy, and psychopharmacology, which can have a significant impact on improving quality of life.^49^

The main limitation of this study is the use of cross-sectional methods, which limit the ability to establish causality between variables and only allow us to identify associations. Another limitation comes from the relatively small number of individuals in specific cancer subgroups, largely due to the overall smaller population in these categories across the country. Therefore, the ability to accurately determine the prevalence of depressive and anxiety symptoms in patients with specific types of cancer may be compromised. However, there are some limitations that need to be taken into account. The generalizability of the findings needs to be reinforced because the study was limited to a particular institution. Moreover, the exclusion of incomplete questionnaires through repetition and collaboration poses a limitation. Despite the constraints, this study is the first of its kind in Qatar and one of the few conducted in the Gulf countries. As a result, understanding of the potential impact of depression and anxiety on cancer treatment in this region has improved significantly.

## Conclusion

According to the study, treatment of mental health issues is essential to enhancing the effectiveness of cancer treatment. Cancer patients may have a higher quality of life and better adherence to cancer treatments if mental illnesses such as depression and anxiety are identified and treated early. Furthermore, 75% of patients reported suffering from depression and 70% from anxiety, according to the study, which showed that these conditions are more common in Qatar than in other countries. Several demographic groups have been linked to higher rates of depression and anxiety, including women, middle-aged adults, those with stage IV cancer, and patients receiving therapies such as radiotherapy and chemotherapy. Furthermore, the study emphasizes the importance of incorporating psychosocial care into cancer treatment plans and the need for more investigation into the root causes of mental health issues in cancer patients.

### Competing interests

The authors have no conflicts of interest to declare.

## Figures and Tables

**Table 1:** Demographic, social and clinical characteristics, personality traits, and quality of life of the participants.

Variables	Values	Proportions (%)

Cancer type		

Blood cancer (hematological malignancies)	99	19.76

Pancreatic/liver cancer	61	12.18

Colon/rectal cancer	47	9.38

Breast cancer	45	8.98

Ovary/cervix/uterus cancer	44	8.78

Trachea/bronchus/lung cancer	40	7.98

Head/neck cancer	39	7.78

Prostate/testis cancer	32	6.39

Kidney/urinary tract/bladder cancer	25	4.99

Brain/central nervous system cancer	17	3.39

Endocrine/thyroid cancer	15	2.99

Others*	37	7.39

Age		

Less than 40 years	129	25.80

40–60 years	301	60.10

Above 60 years	71	14.20

Mean ± SD (years)	47.9 ± 11.4	

Gender		

Male	283	56.50

Female	218	43.50

Socio-ethnic groups		

Arabic group	372	74.20

Non-Arabic group	239	26.80

Family Status		

Single	70	14.00

Married	418	83.41

Divorced	13	2.59

Occupation category		

Housewife	122	24.35

Service worker	102	20.36

Not working	69	13.77

Healthcare professional	41	8.18

Engineer and computer worker	33	6.59

Driver	29	5.79

Business professional	24	4.79

Educator	23	4.59

Office staff	23	4.59

Clerical support worker	13	2.59

Unskilled laborer	16	3.19

Retired personnel	6	1.20

Education		

Illiterate	26	5.19

Primary	40	7.98

Secondary	192	38.32

Tertiary	243	48.50

History of psychiatric illness		

Yes	12	2.40

Family history of psychiatry		

Yes	16	3.19

Family history of cancer		

Yes	166	33.13

Comorbidities		

Yes	206	41.12

COVID-19 infection		

Yes	233	46.51

Hospitalization due to COVID-19		

Yes	41	8.18

Tumor stage		

Stage I	62	12.38

Stage II	110	21.96

Stage III	111	22.16

Stage IV	121	24.15

Not known	97	19.36

Phase of treatment		

Maintenance of a disease-free phase	262	52.30

Relapse or progression phase	193	38.52

Newly diagnosed	46	9.18

Treatment modality		

Combination of chemotherapy and surgery	156	31.2

Chemotherapy	107	21.4

Combination of chemotherapy, radiotherapy, and surgery	95	19.0

Combination of radiotherapy and chemotherapy	28	5.6

Radiotherapy	25	5.0

Cancer-related surgery	22	4.4

Others**	68	13.6

Time since diagnosis		

Mean ± SD (months)	47.9 ± 43.38	

Less than 12 months	99	19.8

12–60 months	312	62.3

More than 60 months	90	17.9


Note: Categorical variables are presented as numbers (%); continuous variables are presented as mean ± standard deviation.

*Includes any cancer types not specifically listed above, such as rare cancers or those without a definitive classification in the categories provided.

**Hormonotherapy, bone marrow transplantation, immunotherapy, and supportive therapy.

**Table 2:** Prevalence of depressive and anxiety symptoms.

Variables	Number (%)

Level of depression	

Normal	125 (24.95)

Minimal	106 (21.15)

Mild	216 (43.11)

Moderate	41 (8.18)

Moderately severe	9 (1.79)

Severe	4 (7.98)

Overall depression	376 (75.05)

Level of anxiety	

Normal	149 (29.74)

Minimal	78 (15.56)

Mild	242 (48.30)

Moderate	21 (4.19)

Severe	11 (2.19)

Overall anxiety	352 (70.25)


**Table 3:** Relationship between depression, anxiety, sociodemographic, and clinical variables.

Variables	Depression		Anxiety	

	*r*_pbi_	*p*	*r*_pbi_	*p*

Cancer type	0.05	< 0.05*	0.04	>0.05

Tumor stage	0.01	>0.05	0.02	>0.05

Date of cancer diagnosis	0.04	>0.05	0.03	>0.05

History of psychiatric illness	0.02	>0.05	0.08	< 0.05*

Family history of psychiatry	0.02	>0.05	0.06	>0.05

Family history of cancer	0.09	< 0.05*	0.07	>0.05

Comorbidities	0.08	< 0.05*	0.01	>0.05

COVID-19 infection	0.04	>0.05	0.04	>0.05

Hospitalization due to COVID-19	0.04	>0.05	0.03	>0.05

Surgical treatment	0.03	>0.05	0.03	>0.05

Exposure to chemotherapy or target therapy	0.06	>0.05	0.09	>0.05

Exposure to radiotherapy	0.05	>0.05	0.10	< 0.05*

Hormone therapy	0.08	< 0.05*	0.05	>0.05

Age	0.01	>0.05	0.06	>0.05

Gender	0.06	>0.05	0.12	< 0.05*

Family status (single/married/divorced)	0.04	>0.05	0.05	>0.05

Education (illiterate/primary/secondary/tertiary)	0.01	>0.05	0.03	>0.05

Occupation	0.05	>0.05	0.04	>0.05

Phase of treatment	0.06	>0.05	0.02	>0.05


Note: The point-biserial correlation coefficient (*r*_pbi_) is an enhanced version of Pearson's correlation coefficient used for measuring the association between binary and continuous variables.

*The bold values indicate statistical significance at the level of *p* < 0.05 (two-sided test).

**Table 4:** Comparison of participant characteristics, depression, and anxiety scores based on the PHQ-9 and GAD-7 scales.

	Depression					Anxiety				

Variables	*n*	%	PHQ-9 (mean ± SD score)	PHQ-9 (median score)	*p*	*n*	%	GAD-7 (mean ± SD score)	GAD-7 (median score)	*p*

Cancer type										

Hematological malignancies	97	26	4.49 ± 3.39	3	>0.05	72	21	4.55 ± 3.56	3	>0.05

Solid tumor	279	74	4.45 ± 3.25	4		280	79	4.46 ± 3.55	3	

Hematological malignancies	97	26	4.49 ± 3.39	3	< 0.05	72	20	4.55 ± 3.56	3	>0.05

Pancreatic/liver cancer	41	11	4.40 ± 3.17	3		49	14	4.46 ± 3.55	3	

Colon/rectal cancer	32	9	5.01 ± 3.69	4		30	9	4.67 ± 3.73	3	

Breast cancer	22	6	5.19 ± 3.83	4		29	9	5.43 ± 4.14	4	

Ovary/cervix/uterus cancer	29	8	4.14 ± 3.20	3		35	10	4.28 ± 4.17	3	

Trachea/bronchus/lung cancer	30	8	4.45 ± 3.25	3		28	8	4.54 ± 3.62	3	

Head/neck cancer	33	9	4.73 ± 3.84	3		36	10	4.83 ± 3.92	4	

Prostate/testis cancer	27	7	4.13 ± 3.43	3		19	5	4.51 ± 3.65	3	

Kidney/urinary tract/bladder	22	6	4.61 ± 4.35	3		17	5	4.41 ± 3.80	3	

Brain/central nervous system cancer	10	3	4.72 ± 3.53	4		8	2	4.86 ± 3.83	4	

Endocrine/thyroid cancer	7	2	3.77 ± 2.72	3		5	2	4.36 ± 3.71	3	

Others*	26	7	4.03 ± 3.21	3		23	6	4.45 ± 4.02	3	

Age										

Less than 40 years	94	25	5.19 ± 3.96	4	>0.05	87	25	5.02 ± 3.92	4	>0.05

40–60 years	217	58	5.09 ± 4.12	4		208	59	4.60 ± 3.68	3	

Above 60 years	65	17	4.88 ± 4.77	3		57	16	4.23 ± 4.09	3	

Gender										

Male	155	41	5.18 ± 4.28	4	>0.05	158	45	4.63 ± 3.83	3	>0.05

Female	221	59	5.02 ± 4.14	4		216	61	4.60 ± 3.72	3	

Socio-ethnic groups										

Arabic group	231	61	5.85 ± 4.74	4	>0.05	214	61	4.53 ± 3.72	3	>0.05

Non-Arabic group	145	39	5.21 ± 4.50	4		138	39	4.87 ± 3.95	3	

Family status										

Single	58	15	4.96 ± 3.65	4	>0.05	57	16	4.26 ± 2.99	3	>0.05

Married	306	81	5.08 ± 4.25	4		284	81	4.67 ± 3.90	3	

Divorced	12	3	6.10 ± 5.57	3.5		11	3	4.91 ± 4.30	4	

Occupation category										

Housewife	90	24	5.28 ± 4.40	4	< 0.05	67	19	4.76 ± 3.81	3	< 0.05

Service worker	70	19	6.17 ± 4.75	5		71	20	4.77 ± 3.76	4	

Not working	55	15	3.21 ± 2.41	3		50	14	3.38 ± 2.57	3	

Healthcare professional	35	9	4.17 ± 3.21	3		31	9	4.77 ± 3.81	4	

Engineer and computer worker	24	6	6.30 ± 4.47	5		20	6	6.24 ± 5.39	5	

Driver	23	6	8.00 ± 4.02	6		22	6	6.59 ± 5.54	5	

Business professional	22	6	6.05 ± 5.33	5		17	5	4.17 ± 2.60	3	

Educator	18	5	3.38 ± 2.42	3		27	8	3.20 ± 2.33	3	

Office staff	17	5	4.35 ± 3.93	3		17	5	5.64 ± 3.62	4	

Clerical support worker	7	2	3.42 ± 2.29	3		12	3	2.83 ± 1.16	2	

Unskilled laborer	11	3	4.09 ± 3.94	3		10	3	4.20 ± 3.91	3	

Retired personnel	8	2	2.33 ± 1.21	2		8	2	3.00 ± 1.41	2	

Education										

Illiterate	23	6	4.78 ± 5.04	4	< 0.05	24	7	4.83 ± 4.16	4	< 0.05

Primary	28	7	8.11 ± 6.01	7		27	8	6.81 ± 5.26	5	

Secondary	148	39	4.78 ± 3.80	4		132	38	4.75 ± 3.57	4	

Tertiary	177	47	4.91 ± 3.88	4		169	48	4.15 ± 3.51	4	

History of psychiatric illness										

Yes	12	3	7.80 ± 5.83	5	< 0.05	196	56	6.25 ± 4.76	5	< 0.05

No	364	97	5.01 ± 4.12	4		156	44	4.58 ± 3.79	3	

Family history of psychiatry										

Yes	14	4	5.73 ± 5.08	4	>0.05	20	6	6.50 ± 3.96	5.5	< 0.05

No	362	96	5.06 ± 4.15	4		332	94	4.54 ± 3.76	3	

Family history of cancer										

Yes	136	36	5.17 ± 4.16	4	>0.05	128	36	4.41 ± 3.64	3	>0.05

No	240	64	5.04 ± 4.22	4		218	62	4.78 ± 3.88	3	

Comorbidities										

Yes	158	42	4.65 ± 4.06	3	>0.05	118	34	4.81 ± 3.75	3	>0.05

No	218	58	5.42 ± 4.26	4		234	66	4.15 ± 3.80	3	

COVID-19 infection										

Yes	184	49	4.88 ± 4.18	4	>0.05	179	51	4.73 ± 3.74	3	>0.05

No	192	51	5.29 ± 4.20	4		195	55	4.50 ± 3.82	3	

Hospitalization due to COVID-19										

Yes	32	9	4.36 ± 3.14	3	>0.05	29	8	4.27 ± 3.09	4	>0.05

No	344	91	5.15 ± 4.27	4		323	92	4.64 ± 3.83	3	

Tumor stage										

Stage I	73	19	5.61 ± 4.31	4	< 0.05	82	23	4.77 ± 3.56	3	>0.05

Stage II	95	25	5.02 ± 4.12	4		104	30	4.38 ± 3.26	3	

Stage III	87	23	4.45 ± 3.61	3		87	25	4.71 ± 3.98	3	

Stage IV	121	32	4.19 ± 3.55	3		79	22	4.81 ± 4.19	4	

Phase of treatment										

Maintenance or disease-free phase	198	53	4.85 ± 3.80	3	>0.05	200	57	4.34 ± 3.39	3	>0.05

Relapse or progression phase	102	27	5.26 ± 5.00	3		100	28	4.80 ± 4.24	3	

Newly diagnosed	76	20%	5.48 ± 3.99	3		74	21	5.12 ± 4.07	3	

Surgical treatment										

Yes	234	62	5.30 ± 4.23	4	>0.05	201	57	4.70 ± 3.78	3	>0.05

No	142	38	4.73 ± 4.10	3		151	43	4.48 ± 3.78	3	

Chemotherapy treatment										

Yes	288	77	5.37 ± 4.41	4	< 0.05	306	87	4.86 ± 3.97	4	>0.05

No	68	18	4.15 ± 3.24	3		46	13	3.80 ± 2.90	3	

Radiotherapy treatment										

Yes	119	32	6.90 ± 4.79	5	< 0.05	106	30	5.69 ± 4.69	4	< 0.05

No	257	68	4.63 ± 3.081	3		246	70	4.19 ± 3.26	3	

Hormone treatment										

Yes	87	23	5.19 ± 4.38	4	>0.05	83	24	5.62 ± 4.23	4	< 0.05

No	289	77	4.77 ± 3.47	4		269	76	4.25 ± 3.01	3	

Time since diagnosis										

< 12 months	79	21	5.44 ± 4.70	4	>0.05	69	20	5.02 ± 3.56	4	>0.05

12–60 months	229	61	5.01 ± 4.59	4		220	63	4.53 ± 3.74	4	

>60 months	68	18	5.00 ± 3.58	4		63	18	4.65 ± 4.30	4	


Note: Student's t-test was used to compare two groups, while analysis of variance F-test was used for more than two groups. A significant difference was found at *p* < 0.05.

*Includes any cancer types not specifically listed above, such as rare cancers or those without a definitive classification in the categories provided.

**Table 5:** Determination and prediction of depression and anxiety risk factors using logistic regression.

	Depression		Anxiety	

Variables	OR^+^ (95% CI)^++^	p*	OR^+^ (95% CI)^++^	p*

Cancer type				

Hematological malignancies	1		1	

Solid tumor	1.02 (0.44, 2.48)	>0.05	1.26 (0.50, 1.88)	>0.05

Age				

Less than 40 years	1		1	

40–60 years	2.43 (1.26, 4.68)	< 0.05	1.27 (0.39, 1.52)	>0.05

Above 60 years	0.23 (0.09, 0.54)	< 0.05	0.61 (0.40, 1.83)	>0.05

Gender				

Male	0.41 (0.19, 0.90)	< 0.05	0.34 (0.15, 0.73)	< 0.05

Female	1		1	

Socio-ethnic groups				

Arabic group	0.41 (0.08, 2.03)	>0.05	1.10 (0.55, 2.44)	>0.05

Non-Arabic group	1		1	

Family status				

Single	1		1	

Married	1.39 (1.51, 3.76)	>0.05	0.16 (0.04, 0.59)	< 0.05

Divorced	5.47 (0.67, 44.62)	>0.05	3.24 (0.03, 27.21)	>0.05

Occupation category				

Not working	1	1		

Clerical support worker	0.30 (0.07, 1.26)	>0.05	0.18 (0.031, 1.01)	>0.05

Taxi driver	0.92 (0.26, 3.19)	>0.05	0.46 (0.11, 1.99)	>0.05

Educator	1.27 (0.32, 4.94)	>0.05	2.39 (1.07, 32.54)	< 0.05

Engineer and computer worker	1.10 (0.32, 3.75)	>0.05	0.73 (0.14, 2.92)	>0.05

Healthcare professional	1.09 (0.33, 3.53)	>0.05	1.29 (0.13, 2.17)	>0.05

Housewife	0.59 (0.21, 1.60)	>0.05	0.43 (0.21, 2.01)	>0.05

Office staff	0.66 (0.16, 0.92)	< 0.05	1.01 (0.20, 4.12)	>0.05

Retired personnel	0.76 (0.11, 5.00)	< 0.05	0.61 (0.40, 33.31)	>0.05

Unskilled laborer	0.71 (0.17, 2.92)	>0.05	1.04 (0.18, 3.46)	>0.05

Government driver	3.06 (0.78, 11.94)	>0.05	2.46 (0.51, 11.81)	>0.05

Service worker	1.01 (0.40, 2.53)	>0.05	1.72 (1.06, 16.99)	< 0.05

Business professional	0.93 (0.26, 3.19)	>0.09	0.61 (0.36, 3.19)	>0.05

Education				

Illiterate	1	1		

Primary	0.37 (0.08, 1.61)	>0.05	0.36 (0.09, 1.44)	>0.05

Secondary	0.22 (0.05, 0.96)	< 0.05	0.42 (0.05, 0.97)	>0.05

Tertiary	0.45 (0.11, 1.88)	>0.05	2.47 (1.08, 5.63)	< 0.05

History of psychiatric Illness				

Yes	1.09 (0.16, 11.42)		0.96 (0.16, 5.69)	

No	1		1	

Family history of psychiatry				

Yes	3.58 (0.35, 36.24)	>0.05	1.29 (0.27, 5.99)	>0.05

No	1	1		

Family history of cancer				

Yes	1.61 (0.98, 2.70)	>0.05	1.23 (0.75, 2.00)	>0.05

No	1		1	

Comorbidities				

Yes	1.63 (1.0, 2.66)	< 0.05	1.20 (0.75, 1.91)	>0.05

No	1		1	

COVID-19 infection				

Yes	1.58 (0.98, 2.53)	>0.05	1.17 (0.74, 1.85)	>0.05

No	1		1	

Hospitalization due to COVID-19				

Yes	0.94 (0.40, 2.22)	>0.05	0.68 (0.30, 1.53)	>0.05

No	1		1	

Tumor stage				

Stage I	1		1	

Stage II	0.76 (0.33, 1.76)	>0.05	0.37 (0.16, 0.86)	>0.05

Stage III	0.57 (0.25, 1.30)	>0.05	0.43 (0.18, 1.01)	>0.05

Stage IV	2.10 (1.154, 3.82)	< 0.05	1.96 (1.08, 3.61)	< 0.05

Phase of treatment				

Maintenance or disease-free phase	0.80 (0.45, 1.41)	>0.05	0.80 (0.43, 1.47)	>0.05

Relapse or progression phase	0.64 (0.34, 1.18)	>0.05	0.50 (0.25, 0.97)	< 0.05

Newly diagnosed	1	1		

Surgical treatment				

Yes	1.46 (0.88, 2.42)	>0.05	1.51 (1.09, 2.44)	< 0.05

No	1	1		

Chemotherapy treatment				

Yes	1.37 (0.78, 2.41)	>0.05	1.51 (0.88, 2.59)	>0.05

No	1		1	

Radiotherapy treatment				

Yes	1.57 (0.90, 2.73)	>0.05	0.79 (0.46, 1.35)	>0.05

No	1	1		

Hormone treatment				

Yes	0.66 (0.37, 1.19)	>0.05	0.94 (0.53, 1.68)	>0.05

No	1	1		

Time since diagnosis				

< 12 months	0.61 (0.27, 1.39)	>0.05	0.86 (0.46, 1.56)	>0.05

12–60 months	1.07 (0.58, 1.98)	>0.05	0.74 (0.41, 1.34)	>0.05

>60 months	1		1	


Note: *p < 0.05, ^+^OR: odds ratio, ^++^CI: confidence interval.
